# Accessibility and Essential Travel: Public Transport Reliance Among Senior Citizens During the COVID-19 Pandemic

**DOI:** 10.3389/fdata.2022.867085

**Published:** 2022-05-23

**Authors:** Ffion Carney, Alfie Long, Jens Kandt

**Affiliations:** The Bartlett Centre for Advanced Spatial Analysis, University College London, London, United Kingdom

**Keywords:** mobility, public transport, smart card data, social exclusion, essential transit users

## Abstract

Using smart card travel data, we compare demand for bus services by passengers of age 65 or older prior to and during the COVID-19 pandemic to identify public transport-reliant users residing in more car-dependent environments—i.e., people who rely on public transport services to carry out essential activities, such as daily shopping and live in areas with low public transport accessibility. Viewing lockdowns as natural experiments, we use spatial analysis combined with multilevel logistic regressions to characterize the demographic and geographic context of those passengers who continued to use public transport services in these areas during lockdown periods, or quickly returned to public transport when restrictions were eased. We find that this particular type of public transport reliance is significantly associated with socio-demographic characteristics alongside urban residential conditions. Specifically, we identify suburban geographies of public transport reliance, which are at risk of being overlooked in approaches that view public transport dependence mainly as an outcome of deprivation. Our research demonstrates once again that inclusive, healthy and sustainable mobility can only be achieved if all areas of metropolitan regions are well and reliably served by public transport.

## Introduction

The COVID-19 pandemic resulted in a significant decline in public transport demand as part of wider changes in travel behavior throughout the UK. The introduction of numerous lockdowns and restrictions in 2020 led to an increase in people working from home and the replacement of usual out-of-home activities with online equivalents (Department for Transport, 2021). Whilst restrictions have since eased in the UK, a simple return to pre-pandemic travel behavior seems unlikely. In order to support public transport authorities and operators in providing an inclusive public transport system that meets the needs of users, the field requires continued research into changes in public transport patronage by different population groups throughout the COVID-19 pandemic.

Due to their vulnerability, older, senior citizens were advised particularly strongly to isolate, or “shield”, and as a result they may have been more reluctant to return to public transport services once restrictions eased. Restricted mobility arising from this can limit an individual's ability to perform both essential and non-essential activities, which negatively influences social participation, quality of life and wellbeing (De Vos, 2020).

This study focuses on the use of bus services by concessionary passengers of age 65 or older in the West Midlands Combined Authority (WMCA) during the COVID-19 pandemic in 2020. Our aim is to harness big data—linked smartcard transactions—to identify and characterize particularly vulnerable *essential transit users*, i.e., individuals who appear to depend on public transport for essential trips while living in low accessibility, more car-dependent environments. This particular configuration of vulnerability often remains concealed in aggregate, lower public transport demand that “naturally” occurs in more car-dependent location. We hypothesize that lockdowns with requirements to restrict all travel to but essential trips can uncover hitherto less considered types of vulnerability, specifically that of public transport reliance in low accessibility areas. In view of the issues around transport and social exclusion, understanding this type of vulnerability is important for the delivery of inclusive transport systems during and after the COVID-19 pandemic.

## Literature Review

Since the outbreak of COVID-19, there has been extensive research into the impact of the pandemic on the level and types of travel demand. Most studies have focused on aggregate demand across public transport networks (see Jenelius and Cebecauer, 2020; Zhang et al., 2021), however individual-level studies are emerging (Kopsidas et al., 2021; Przybylowski et al., 2021).

### Globel Trends in Travel Demand

The outbreak of COVID-19 in early 2020 prompted the introduction of numerous lockdowns and restrictions to curb the spread of the virus in many countries across the globe. These measures primarily aimed to restrict the movement of people, for example through requirements to work from home or bans on travel for non-essential trips (Institute for Government, 2021). As a result, significant travel behavior adaptations have been observed across the globle in a sector normally considered resistant to change (Marsden and Docherty, 2021). Travel demand shifted from physical commutes to home working, there was greater adoption of e-shopping and home delivery services, and an increase in active travel, i.e., walking and cycling, and changes in ride-hailing use (Matson et al., 2021), whilst demand for public transport services experienced a major decline. This decline was most significant at the early stages of the COVID-19 outbreak and recovered to varying extents once restrictions were eased (Kim and Kwan, 2021).

Yet changes in public transport demand within population groups were not homogenous. Poorer and more deprived populations used public transport more often during the pandemic and differences were also evident between ethnic groups, age groups and residential locations (Liu et al., 2020; Almlof et al., 2021; Kim and Kwan, 2021). As well as impoverished and working-class populations, communities with higher proportions of essential workers and vulnerable populations, including those from minority ethnic groups, females and people aged over 45, maintained higher levels of public transport patronage during the pandemic (Liu et al., 2020).

Differences have also been observed in the recovery of public transport demand when restrictions were eased during the summer months of 2020. Individual-level studies identified that return to public transport was influenced by whether individuals were frequent public transport users prior to the pandemic and by age, with frequent users and younger people more likely to return to public transport services post-pandemic (Kopsidas et al., 2021). Of those that have said they will not continue to use public transport services one of the most common reasons was that they felt these services will never be safe (Ravensbergen and Newbold, 2020; Przybylowski et al., 2021). Other research points toward mode choice, and accessibility (Palm et al., 2021), being the main factor for reduced demand, though this does not rule out a return to pre-pandemic levels.

### Inequalities in Travel Demand

There is evidence that the COVID-19 pandemic has exacerbated existing inequalities within transport and social participation. The lockdowns and restrictions that were introduced had a differential impact on the mobility of populations, with unequal movement observed by both neighborhood deprivation and socioeconomic status (Campbell et al., 2021). In terms of the spatial structure of travel demand, Kar et al. (2021) found in the United States that diffused travel patterns of individuals of higher and moderate socioeconomic status were found to have become more localized during the pandemic, while the pre-existing localized travel patterns of low socioeconomic status populations became diffused. The curtailing of movement through lockdowns and restrictions on travel appeared to exacerbate underlying social and spatial inequalities.

The decline in public transport use during the COVID-19 pandemic was partly the result of a shift toward private transport, i.e., the car, walking and cycling (Matson et al., 2021). An immediate return to pre-pandemic travel behaviors is deemed unlikely in the literature, and the modal shift thus may negatively affect those that are already vulnerable to transport disadvantage, such as those with mobility impairments and disability-restricted groups (Teuton et al., 2020).

For those that are unable to participate in active modes of transport or lack private motor vehicle transport options, access to public transport services can be vital for mobility and inclusion. These populations can be described as *public transport captives*, i.e., public transport is the only mode available to them (Beimborn et al., 2003). Whilst this does not necessarily equate to transport disadvantage, as public transport services can be both accessible and efficient, this lack of mode choice does imply that these populations are more vulnerable to transport disadvantage and subsequent social exclusion. Older people have been found to be particularly vulnerable (Andrews et al., 2012; Key and Culliney, 2016). The impact of reduced capacity and use of public transport services are likely to be felt the most by those that are already transport disadvantaged (Teuton et al., 2020; Vickerman, 2021).

### Public Transport Reliance in Car-Dependent Environments

In the context of the pandemic, He et al. (2022) have introduced the term *essential transit riders* to describe the group of users who continue to use public transport as regularly during the pandemic as they did before. In their paper, socioeconomic circumstance and ethnicity is shown to be a significant factor in being an *essential transit rider*. The notion of *essential transit user* implies a high degree of reliance of public transport for any reason, be it structural, spatial, economic or by some degree of choice (He et al., 2022).

This discussion links to long-standing debates of transport captivity. Beimborn et al. (2003) divided travelers into two groups: choice users and captive users. Choice users were defined as those who select the mode of transport they use as they view this option as superior to others, whereas captive users were defined as those who only have one travel option. Public transport captives can therefore be defined as those for which public transport is the only available or viable mode choice. The issue of public transport dependence and mode choice amongst the older population has also been the subject of survey research (Ravensbergen and Newbold, 2020), though through a qualitative approach.

We build in the notion of *essential transit users* and focus in particular on those passengers that exhibit these characteristics while living in areas of low public transport accessibility. Areas of low public transport accessibility reflect an environment of higher car dependence and such areas can deny access to essential goods and services, leading to social exclusion among those with lack of access to alternatives (Levitas et al., 2007; Lucas, 2012). We refer to those passengers who lived in low accessibility areas and continued to use public transport at the same frequency as before as *access deprived essential transit users*. We argue that this specific configuration of vulnerability—high public transport reliance in low accessibility areas—must be understood if all forms of mobility needs are to be considered in pressing debates of how inclusive transport systems should be provided as societies deal with the consequences of the pandemic (Vickerman, 2021).

## Research Design

Most big data approaches to understanding mobilities in the COVID-19 pandemic focus on aggregated levels of information, for example total demand by mode, or area or station. Sources used are of uncertain socio-demographic provenance, and demand is often estimated in aggregate terms by mode or time (Cartenì et al., 2021; Rodríguez González et al., 2021). Big data, non-survey studies that successfully link individual trips to social and geographical characteristics at the micro level remain rare.

### Study Area

**Figure 1:**
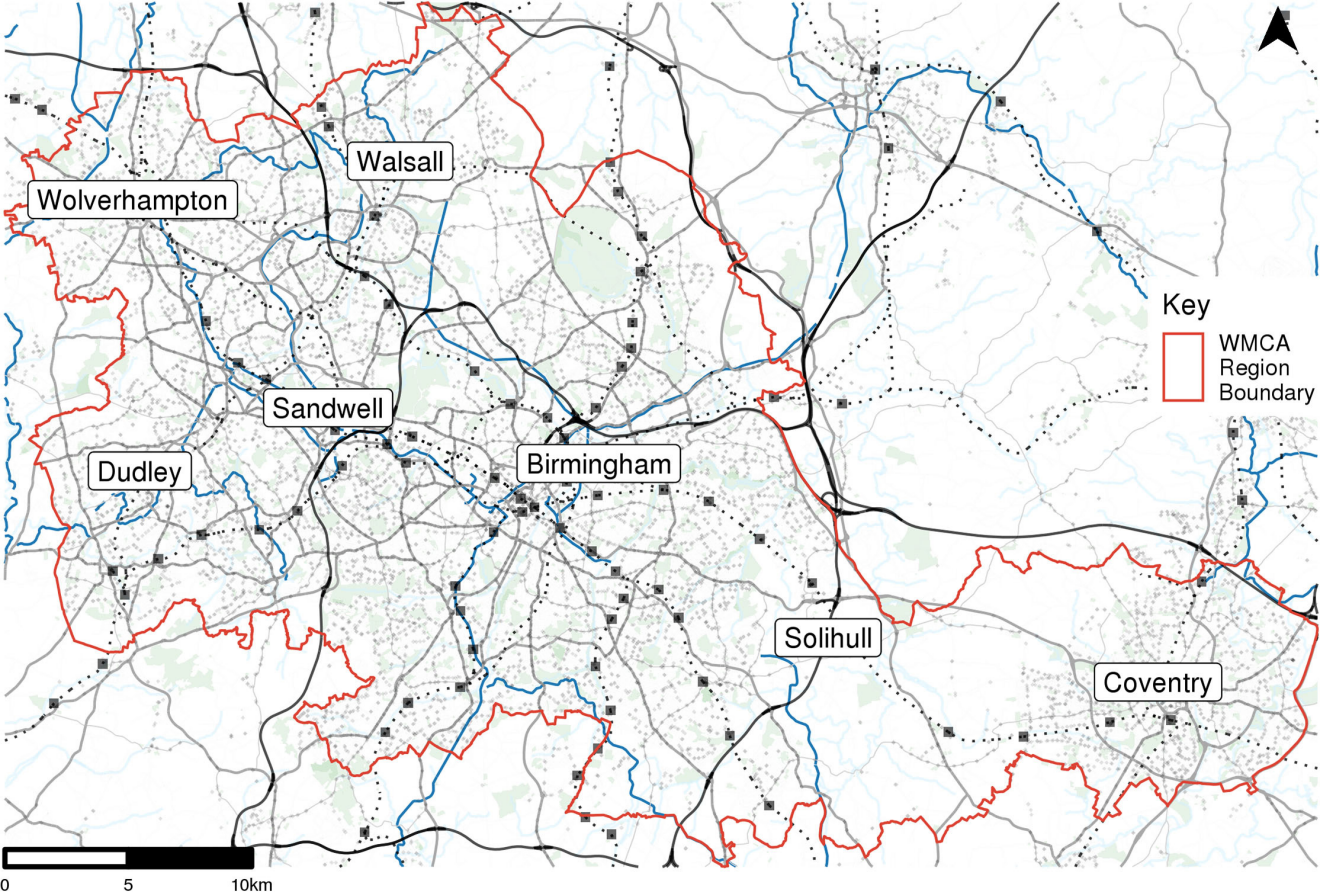
The study region, West Midlands Combined Authority (WMCA), and its seven constituent local authorities.

The West Midlands Combined Authority (WMCA) covers a large urban area within the West Midlands region of England in the UK with a total population of around 2.9 million. This is a densely populated polycentric conurbation with the major city of Birmingham in the center, Wolverhampton to the west and Coventry to the east. The combined authority itself is made up of seven local authorities that share responsibility for public transport through the organization Transport for West Midlands. Some of these contain individual cities, such as Birmingham and Coventry, and others, particularly Dudley and Sandwell, contain many smaller town centers linked by continuous urban areas. Solihull also contains a large green belt area between the town of Solihull and the outskirts of Coventry. Figure 1 shows a map of the WMCA and each of the local authorities.

### Linking and Processing Travel Smart Card Data

We analyse smart card transactions linked to anonymised passenger databases pertaining to older concessionary smart card holders on the bus network in the WMCA. Older concessionary cardholders are those that are over state pension age and therefore eligible for the English National Concessionary Travel Scheme (ENCTS). ENCTS permits free bus and rail travel after 9.30 a.m. on the entire network in the region. Our study period spans the entire calendar years of 2019 and 2020 covering travel before and during the COVID-19 pandemic.

The anonymised passenger database contains the age, gender and ethnic group of cardholders. Using card IDs, it is possible to link smart card transactions to demographic attributes. Ethnic group is recorded in five categories—White, Black, Asian, Mixed and Other. In this analysis we group the age into 5 year bands to simplify analysis. Gender is recorded as Female or Male.

The total cardholder register holds over 400,000 individuals. First the data was filtered for active users, i.e., cardholders that made at least one transaction in 2019. Then the data was filtered for cardholders with complete demographic data. By complete demographic data we mean that all of the three fields of age, gender and ethnicity that have a specified value. Some fields have “NA”, or “Unspecified”, and therefore records with these unspecified values are excluded from the analysis. This resulted in a dataset containing 221,631 cardholders.

### Segmenting Passengers and Measuring Return to Public Transport

Cardholders were firstly grouped by their frequency of bus use in 2019 based on average weekly boardings. To only include cardholders that were active throughout 2019, “joiners” and “leavers”, i.e., cardholders that either joined the ENCTS or left the scheme, were identified and excluded from the analysis. These were defined as cardholders that made no transactions in the first 3 months of 2019 (joiners) or the last 3 months of 2019 (leavers). Of the 221,631 cardholders, 69,570 were identified as either joiners or leavers. This resulted in a final dataset containing 152,061 cardholders that were active throughout 2019.

The average number of weekly boardings over 2019 was calculated for each of the remaining cardholders, removing the first and last week of the year to only include full weeks. Analyzing the distribution of average weekly boardings, cardholders were grouped into five segments representing differential frequencies of bus use: ≥0 and <1, ≥1 and <2, ≥2 and <4, ≥4 and <7, and ≥7 average weekly boardings.

This segmentation was used to determine whether a cardholder returned to their average 2019 activity levels post-COVID outbreak. As the focus of this study is on essential public transport users, and therefore individuals that used the bus network to undertake essential activities, this analysis focused on those that returned to the bus network before 15th June which was the date that non-essential shops were re-opened. Firstly, the number of boardings made by each cardholder were calculated for each week in the period from 23rd March to 15th June 2020, representing the period during which non-essential shops were closed and travel was restricted to essential trips only. A cardholder was determined to have returned their pre-COVID activity levels when their 4-week rolling average reached the level of their pre-COVID segment, i.e., their average bus use during this period of 2020 equalled their average use during 2019. This allowed us to identify those that returned to using the bus network regularly for essential trips as well as the date at which they returned to their pre-COVID activity levels, defined as the first day of the last week of the 4-week rolling average.

### Estimating Accessibility

To identify the low accessibility areas we derive a measure of accessibility to food retail by walking and public transport. We calculate the time taken to access food retail locations, including both retail areas and supermarkets, via the public transport network and walking. Journey times are calculated over the course of a typical weekday to provide an average journey time, and therefore accessibility measure, for each area. For this analysis, the accessibility metric refers to the average travel time from Output Area (OA) centroids to the nearest 3 retail areas via the bus network. This gives an indication of the level of public transport provision for a given area.

**Figure 2:**
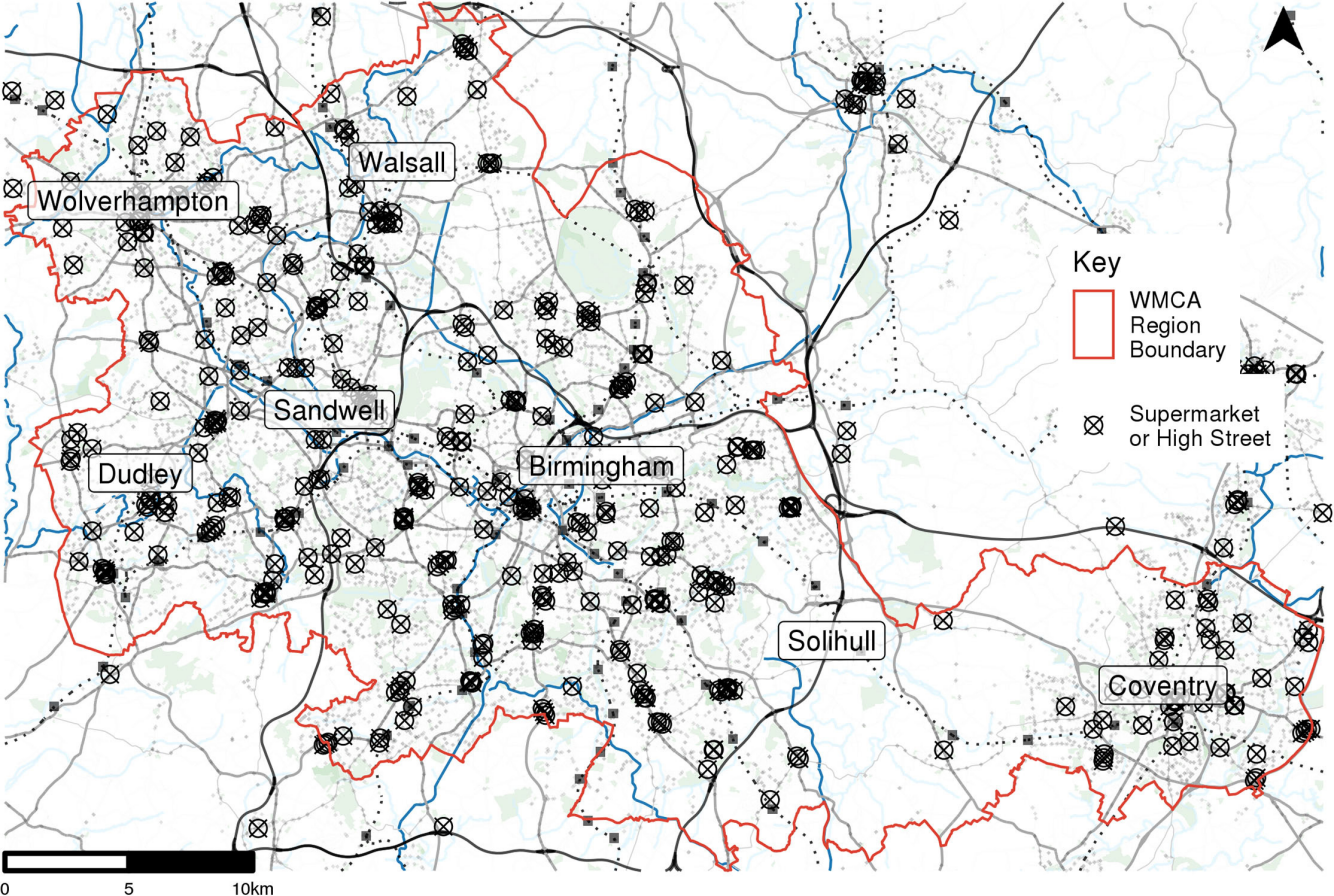
High street and supermarket centroid locations.

Supermarket locations and high streets were selected as the essential retail locations they are areas and facilities that attract essential trips. Supermarket locations were determined using Geolytix Supermarket Retail Points data (Geolytix, 2021). These data were filtered to only contain supermarkets that were open during the study period and only those that with a size band of “medium” or larger. As this dataset only contains major brand supermarket locations, high street location data were also included to account for trips to smaller food shops and other essential facilities, such as pharmacies. High street locations were extracted as centroids from CDRC retail center boundary data (CDRC, 2021) for all retail areas larger than “small local centres”. All supermarket and high street locations within the WMCA area and within a 10 km buffer were included in the analysis. This resulted in 52 high streets and 442 supermarkets, the locations of which are shown in Figure 2.

**Figure 3:**
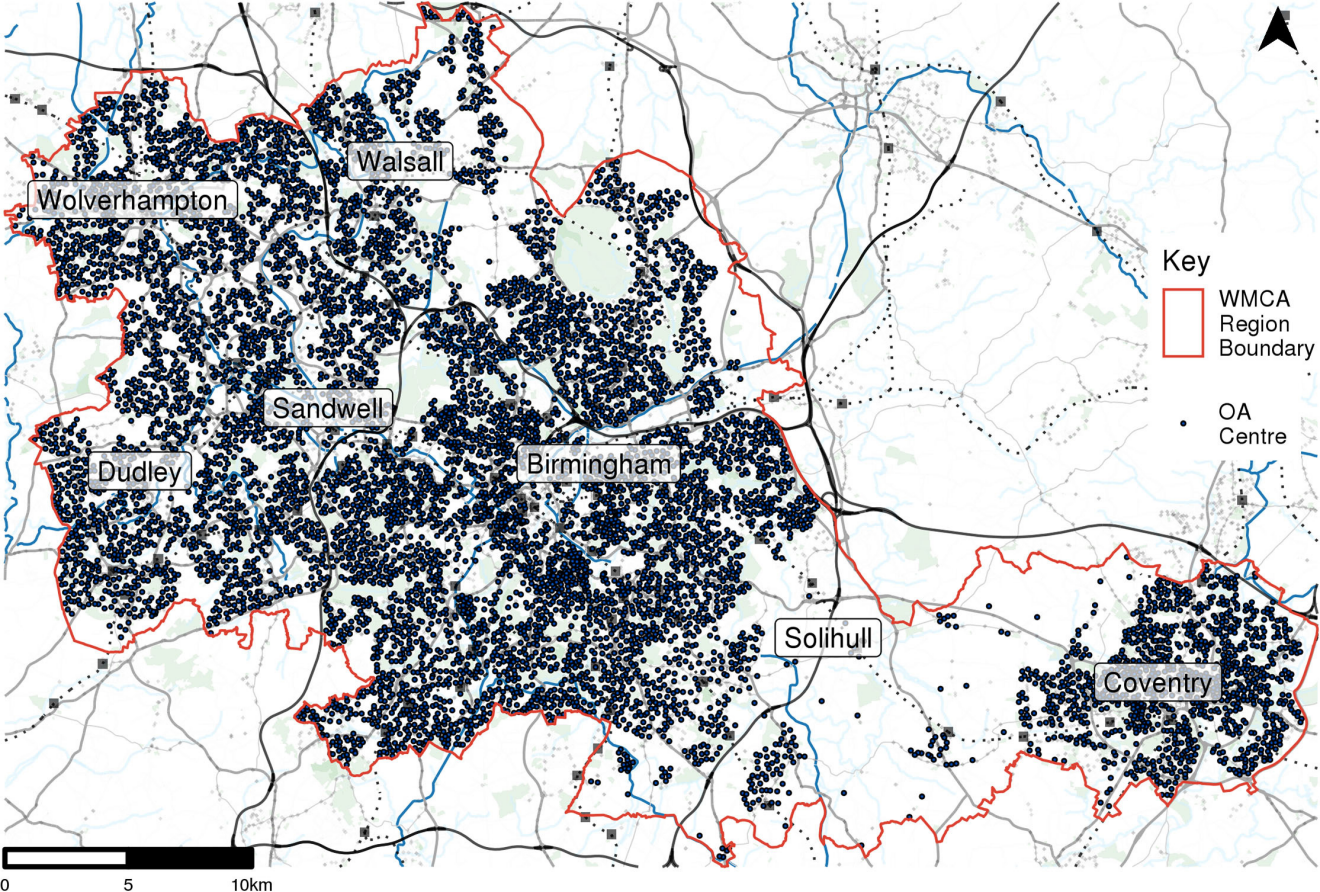
Centroid locations of UK census output areas.

OAs are the lowest geographical level at which census estimates are available and were selected as the origins of the travel time estimations to allow for linkages to census data. An OA has an average population of 309 in England and Wales (Office for National Statistics. Census Geography n.d.). Using the lowest geographical level means that the population weighted centroids are more numerous in more densely populated areas, and that each unit has an approximately similar population size. These are designed to fit within the hierarchy of other census geographies and administrative boundaries so can be aggregated to larger geographies, such as Lower Super Outputs Areas (LSOAs), allowing for linkages to more aggregated datasets such as Index of Multiple Deprivation (IMD) data. Figure 3 shows the distribution of these 8,468 OA centroids in the West Midlands.

To calculate travel times between OA centroids and retail areas, bus timetable data is downloaded from Traveline (Traveline, 2021) in TransXChange format and converted to General Transit Feed Specification (GTFS) format using the CLI tool *transxchange2gtfs* (Norton, 2018). These timetable data are then combined with road routing data from OpenStreetMap (2021) to create the graph object that OpenTripPlanner (2021) uses to calculate travel times. The routing algorithm includes the time taken to walk to a transport stop, the time spent waiting at the stop and the transfer time between services if more than one service is needed.

**Figure 4:**
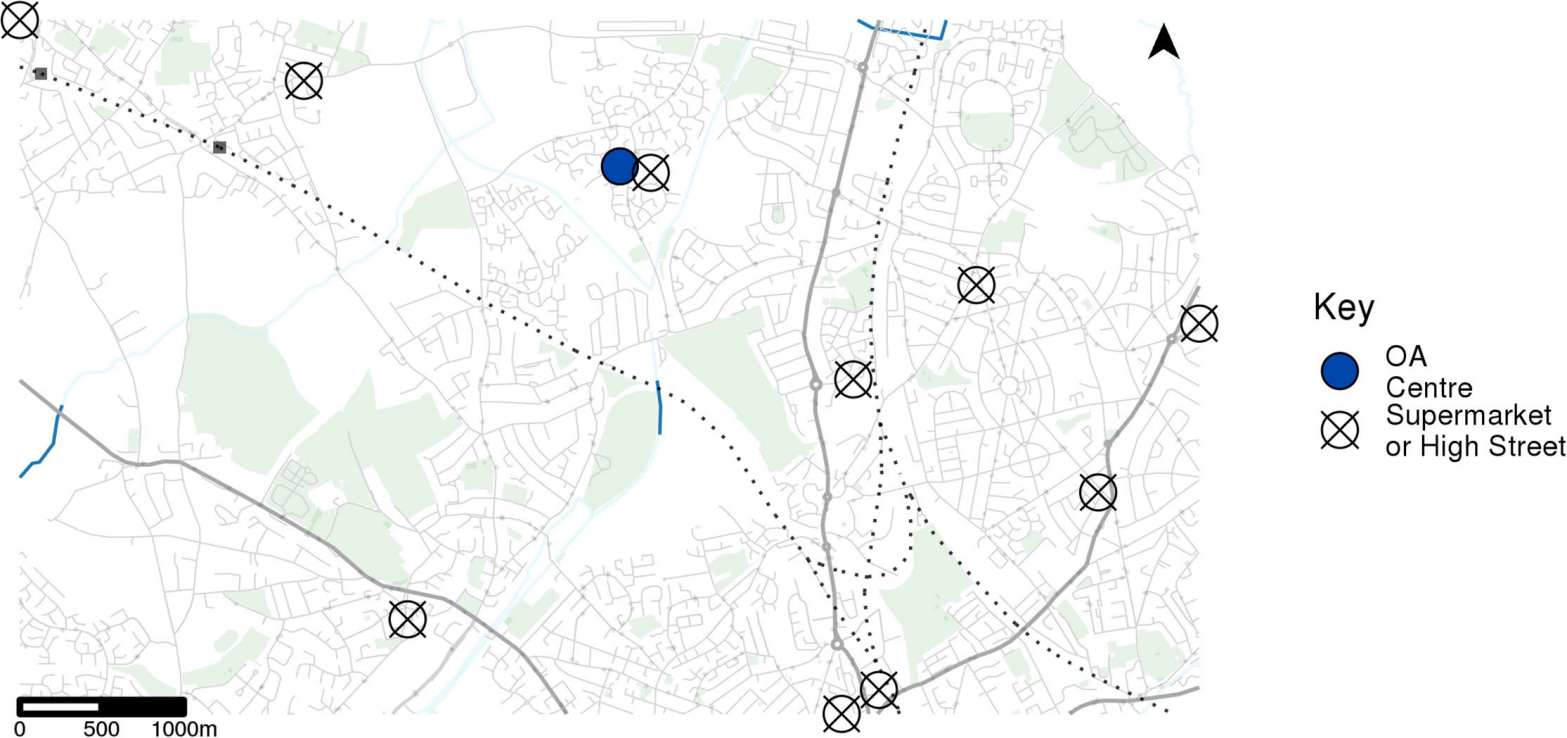
Example census output area (OA) centroid and 10 nearest retail location centroids.

To obtain an average accessibility measure for each OA, the 10 nearest retail locations are selected using the Euclidean distance. The shortest travel time between the OA centroid and each of its 10 destinations walking, or walking and using public transport services is calculated. The average travel time is then determined by taking the average of the three lowest travel times. For this analysis, travel times were calculated at 10-min intervals between 8 a.m. and 8 p.m. on 7th April 2020 with these then averaged to give a typical travel time measure for a weekday for each OA. Figure 4 shows an example OA centroid and 10 nearest retail locations.

### Identifying Essential Public Transport Users

Combining the segmentation and accessibility analyses allows for the identification of *access deprived essential transit users*. These are defined as cardholders that returned to their 2019 activity levels by 15th June 2020 and resided in low accessibility areas. Low accessibility areas refer to areas with an average travel time of over 15 min to the 3 closest retail areas, calculated in Section Segmenting Passengers and Measuring Return to Public Transport. Fifteen minutes was chosen as the threshold, as 10 min is below the median average travel time for all the OAs in the study area. Likewise raising the threshold to over 20 min left only 734 individuals within the sample, and for many of the demographic categories too few to carry out statistically rigorous analysis.

We implemented a multi-level logistic regression model to analyse the characteristics of these *access deprived essential transit users*, including their demographic attributes and pre-COVID bus usage as well as deprivation and car ownership levels in the areas they reside. A multi-level model was chosen so that both individual characteristics, included in the cardholder data, could be included alongside area-level characteristics. The dependent variable is “essential public transport user” recorded as either “yes” or “no” indicating whether a cardholder was identified as an *access deprived transit user*. The odds ratio is calculated for each variable to measure the degree to which a change in category or value is associated with the likelihood of being a transport captive, with respect to the reference category.

## Results

### Passenger Segments and Frequency of Boardings

The segmentation of cardholders based on their average weekly bus boardings in 2019 resulted in five groups with differential bus use frequency, shown in Table 1.

**Table 1:** Bus use frequency segments.

**Segment**	**Weekly boardings (average)**	**Number of cardholders**
1 – Rare	≥0 and <1	52,464
2 – Infrequent	≥1 and <2	23,998
3 – Frequent	≥2 and <4	25,902
4 – Regular	≥4 and <7	21,052
5 – Daily	≥7	28,645

These segments were selected as they encapsulate a range of bus usage behaviors, from those that rarely use the bus network to those that, on average, use the bus network at 7 or more times in a week. Except for segment 1, there are a similar number of cardholders in each segment.

**Figure 5:**
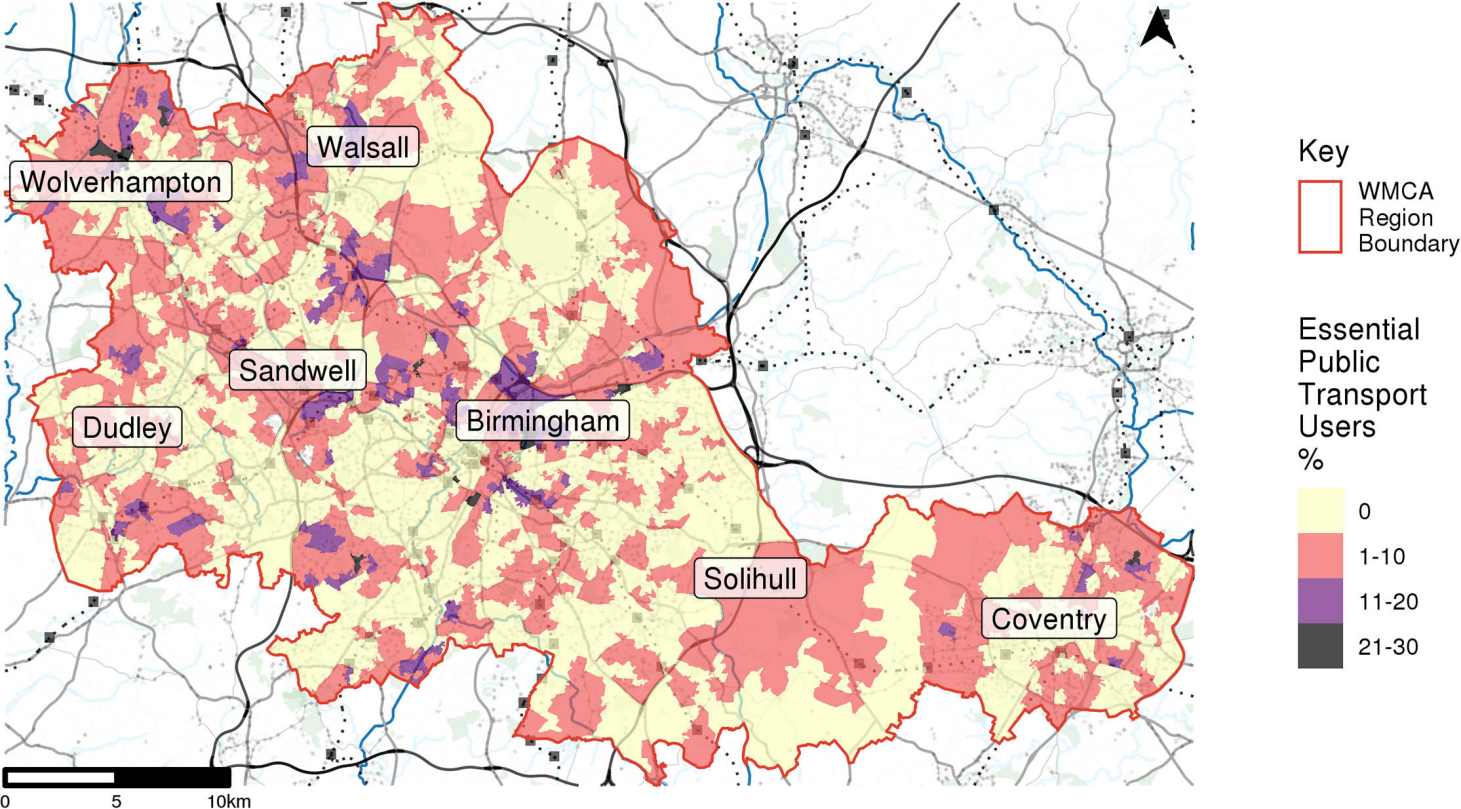
Number of access deprived transit users at LSOA level.

Of the 152,061 cardholders included in this analysis, 62,125 were found to have returned to their pre-COVID activity levels and 3,516 of these cardholders were found to reside in low accessibility areas. These were identified as vulnerable essential public transport users, the residential locations of which are shown in Figure 5.

Essential public transport users are distributed throughout the study area, with few areas containing counts of more than 10. As would be expected with only those residing in low accessibility areas, most of these cardholders reside in rural and suburban areas, in particular throughout Solihull.

### Characterizing Access Deprived Essential Transit Users

To understand the characteristics of these users, we ran multilevel logistic regression with the binary outcome of *access deprived transit user* and explanatory variables related to demographics, socioeconomics, pre-COVID activity and geographic context.

Table 2 shows that several independent variables have a significant relationship with being an *access deprived essential transit user*. Regarding individual-level variables, younger cardholders are more likely fall into this category than older cardholders, with those aged >85 almost 50% less likely than those aged 66–70. As well as reliance on public transport services, this also likely reflects a greater willingness to return to services. On the flipside, these results may indicate that there are transport captives within our population, especially in the oldest age segments, who are unable to return to public transport and thus suffer from exclusion during the pandemic. We will return to this point below.

**Table 2:** Multilevel logistic regression with “access deprived” (“yes” and “no”) as the dependent variable.

**Independent variable (reference category)**	**Access deprived essential transit users**			
	**Estimate**	**Std. error**	**Odds ratio**	**sig**.
**Individual-level**				
**Age (66–70)**				
71–75	−0.341	0.048	0.71	^***^
76–80	−0.498	0.052	0.61	^***^
81–85	−0.571	0.059	0.57	^***^
>85	−0.673	0.073	0.51	^***^
**Gender (female)**				
Male	0.459	0.036	1.58	^***^
**Ethnic group (white)**				
Asian	−0.373	0.065	0.69	^***^
Black	0.396	0.073	1.49	^***^
Mixed	0.114	0.222	1.12	
Other	0.121	0.102	1.13	
**Frequency (cont.)**				
Segment	0.663	0.015	1.94	<2e-16 ^***^
**Area-level (LSOA)**				
IDAOPI (cont.)				
IDAOPI Decile	−0.144	0.032	0.87	8.84e-06^***^
**Car ownership (cont.)**				
Percentage car owners	0.025	0.005	1.02	7.57e-06 ^***^
*R*^2^ = 0.49				

Signif. codes: 0 ‘***' 0.001 ‘**' 0.01 ‘*'.

Male cardholders are significantly more likely to be *access deprived essential transit users* than female cardholders which may also reflect the higher percentage of older men that are still in employment than women, and therefore use the bus network for commuting purposes. Comparing between ethnic groups, those of an Asian ethnic background are significantly less likely to be identified in our categorization than White cardholders whilst those belonging to Black ethnic groups are significantly more likely. This implies the presence of transport inequalities between ethnic groups.

The segments constructed in Section Linking and Processing Travel Smart Card Data correspond to the frequency of bus use in 2019, with Segment 1 containing those that used the bus rarely and Segment 5 those that were daily bus users. The significant positive relationship therefore indicates that continued public transport use in low accessibility areas is expressed through more frequent bus patronage prior to the pandemic. These frequent bus users are more likely to return to bus services post-COVID and return their 2019 activity levels. With these identified as essential public transport users also residing in low accessibility areas, these cardholders are likely to be particularly vulnerable to transport disadvantage.

The area-level variables, IDAOPI Decile and car ownership, also show significant associations with *access deprived essential transit users*. An increase in IDAOPI Decile, indicating a decrease in deprivation, has a significant negative relationship with categorization. This means that cardholders residing in more deprived areas are more likely to be *access deprived essential transit* users. Yet, they were also more likely to reside in areas with higher car ownership rates. This result reflects the lower levels of public transport service in less dense, often suburban areas, which also have higher levels of car ownership and fewer opportunities to satisfy essential trips by walking. Such areas are often thought of as more affluent, but this analysis reveals that they can nevertheless be home to transport disadvantage (Allen and Farber, 2021), which would be masked by an analysis of social deprivation alone.

## Discussion: Identifying and Addressing Access Deprived Essential Transit Users in Later Life

Linked smart card data are longitudinal data that cover an entire population group of the over 65s on one mode of transport. This resource allows us to study specific types of vulnerability, adding to the extensive survey-based research in this area. Traced against the policy intervention of lockdowns, we find that *access deprived essential transit users* overcome significant barriers to their mobility in terms of time and distance to activities, goods, and essentials during non-pandemic times even though these barriers are augmented during the pandemic by the significant restrictions on their activity from UK government lockdowns. Whilst we cannot explain the motives, our research can explore the revealed activity and quantify the demographic makeup of *access deprived essential transit users*.

### Access Deprived Essential Transit Users Among Older Citizens in the WMCA

The results of this study suggest that there are a significant number of WMCA residents who rely on public transport services for making essential trips yet reside in areas with low levels of public transport provision. COVID-19 has the potential to exacerbate existing inequalities and lead to stronger selectivity of mode use in the region, in particular an increase in active and private vehicle transport use. For the identified group this could lead to increased disadvantage if these populations are unable to make use of public transport services due to reduced capacity and provision.

There is a call to capitalize on larger step changes as institutions are ready to make larger than usual changes due to the “mega-disruption” of the COVID-19 pandemic (Marsden and Docherty, 2021). The research in this paper shows how existing public transport big data infrastructure can be used to better understand COVID-19 pandemic disruptions, but also the existing patterns and inequalities the disruption has uncovered.

Vulnerable transport captives are distributed throughout the metropolitan region, particularly in rural and suburban areas. Prioritizing improved public transport services in the areas identified as having high numbers of *access deprived essential transit user* could go some way to minimizing the potential disadvantages and inequalities that COVID-19 has caused or exacerbated. Equally, alternative public transport services, such as on-demand and ring-and-ride transport, may be viable options for targeting essential public transport users residing in low accessibility areas. Since many of these services increasing relie in digital technologies, efforts need to be made to overcome the digital divide which remains pronounced among older citizens (Carney and Kandt, 2022).

The results of the multilevel logistic regression expand on this further. An increase in the relative deprivation of an area is associated with an increase in the likelihood of a resident being an *access deprived essential transit user*. This suggests that any changes to public transport services in deprived rural and suburban areas would have a significant effect on users within these geographies. Similarly, higher car ownership in an area is associated with higher proportions of ENCTS users being *access deprived essential transit users*. This highlights the potential for a move toward private motor vehicle transport to exacerbate existing transport inequalities.

In terms of the geographical distribution, we find that *access deprived essential transit users* are spread across the entire region with some evidence for the long-term trend of *suburbanization of transport poverty* (Allen and Farber, 2021), which has also been observed in North America. Here we may observe signs of a common challenge for car-dependent societies and nations with extensive areas of deprived suburban and rural communities. Whilst this group may be a minority of the overall population, they are present across a variety of administrative boundaries, towns and cities within the study region. Targeting this population with better services may be economically inefficient for privatized bus services, but it is an important step toward proving an inclusive and sustainable transport network during and after the pandemic.

### “Big” Smartcard Datasets to Identify Vulnerability

Whilst we cannot reliably speculate on the possibility of mode choices for the individuals within our study, we can record their transport use alongside the level of access to essential goods and services via the public transport network. Although our research does not confirm choice availability as readily as survey-based research, we can observe that *access deprived essential transit users* are overcoming inequitable circumstances to go about essential activities during a national lockdown.

In view of the risk of widening inequalities in transport and mobility post pandemic (Blundell et al., 2020; Bohman et al., 2021), gaining detailed insights into public transport usage during the COVID-19 pandemic is a crucial step to identify interventions that minimize the risk of social exclusion faced by vulnerable and older populations. Travel smart card data provide a powerful big consumer data source for understanding the impacts of COVID-19 and assessing the future of public transport services.

Moreover, the processing of individual-level smart card data during the pandemic as part of a quasi-experimental research design presented an opportunity to focus solely on those individuals that rely on public transport services for making essential trips. We were thus able to identify population groups, both in terms of their demographic and socioeconomic attributes and the areas in which they reside, that rely on public transportation but are potentially underserved by the current transport network. Analyses utilizing smart card data can provide transport authorities with more detailed insights into transport inequalities and the future targeting of transport investment.

To achieve this, some technical choices had to be made. This study defined *access deprived essential transit users* as those users that returned to their pre-pandemic activity levels during the period when only essential travel was permitted. While this was effective in identifying cardholders that quickly returned to their usual travel behavior, it does not account for non-essential trips that were being undertaken pre-pandemic. Essential trips were only able to be identified for the period of 23rd March to 15th June 2020, as all other trips were prohibited, and therefore bus activity during 2019 may have been for both essential and non-essential purposes.

It is therefore likely that additional cardholders may have returned to their pre-pandemic levels of essential trip making, but their overall activity levels may have remained below this pre-pandemic level due to the exclusion of non-essential trips in 2020. Due to the lack of information regarding trip purpose, it was not possible to separate essential and non-essential activity during 2019. Future research could expand upon this study to focus on the varying degrees to which different population groups returned to bus services with the acknowledgment that non-essential activity would not have been expected to have resumed during the 2020 study period.

Additionally, defining *access deprived essential transit users* as those that returned to public transport services may exclude the most vulnerable populations. These may have been high frequency bus users pre-pandemic but have not returned to public transport services, not because they have access to alternative transport options, but because they do not feel safe or comfortable doing so. Although identifying these populations is beyond the scope of this research, it is important to note that a non-return to public transport could also be indicative of transport disadvantage.

Nevertheless, the approach of using a big dataset with large coverage over the population addresses many of the shortcomings of voluntary survey-based approaches and adds to the literature by providing a quantitative mobility-based approach to understanding the linked issues of accessibility, transport captivity and public transport demand.

## Concluding Remarks

The availability of smart card data for periods both prior to and during COVID-19 has presented new opportunities for conducting detailed analyses of travel behavior during a pandemic. Additionally, restrictions on travel presented an experimental context for the analysis of essential trips. This has allowed for a detailed analysis of essential travel through the identification of *access deprived essential transit users*. The combination of a large sample size along with the ability to identify essential trip-making means this study can uncover less studied types of public transport reliance. The insights produced from this study can aid in the identification of those that are particularly vulnerable to changes in travel behavior and provision brought about by COVID-19, as well as providing detailed information on which population groups and areas should be targeted with future transport investments and interventions.

## Data Availability Statement

The datasets presented in this article are available via secure services provided by the ESRC Consumer Data Research Centre (CDRC). Data can be obtained on application at data.cdrc.ac.uk.

## Ethics Statement

The studies involving human participants were reviewed and approved by UCL Research Ethics Committee. The patients/participants provided their written informed consent to participate in this study.

## Author Contributions

FC, AL, and JK conceived of the central concept and interpreted the results. FC and JK decided the passenger boarding segmentation. AL organized and simplified the mobility data and generated the accessibility data structures and results. FC researched and wrote the literature review. AL and FC tested the transport captive parameters and the outputs. FC implemented the logistic regression model, and interpreted the results with contributions from JK and AL. All authors contributed to the article and approved the submitted version.

## Funding

Parts of this research were funded by the ESRC-Canada AI initiative (ES/T012587/1) and an ESRC Ph.D., studentship co-sponsored by Transport for West Midlands (TfWM) (ES/P000592/1). The data were obtained under an academic user licence from the ESRC Consumer Data Research Centre (CDRC, www.cdrc.ac.uk).

## Author Disclaimer

The authors are fully and solely responsible for all analysis, results and interpretation; and nothing in this manuscript should be deemed as reflecting the views of TfWM.

## Conflict of Interest

The authors declare that the research was conducted in the absence of any commercial or financial relationships that could be construed as a potential conflict of interest.

## Publisher's Note

All claims expressed in this article are solely those of the authors and do not necessarily represent those of their affiliated organizations, or those of the publisher, the editors and the reviewers. Any product that may be evaluated in this article, or claim that may be made by its manufacturer, is not guaranteed or endorsed by the publisher.
